# What works in real-world implementation? Practice-based insights from the ENJOY IMP-ACT project promoting physical activity among older adults

**DOI:** 10.1093/heapro/daag120

**Published:** 2026-08-07

**Authors:** Pazit Levinger, Marcia Fearn, Bronwyn Dreher, Adrian Bauman, Sze-Ee Soh, Natasha K Brusco, Andrew Gilbert, Rachel Muoio, Elissa Burton, Keith Hill

**Affiliations:** National Ageing Research Institute, Royal Melbourne Hospital, Melbourne, Victoria 3050, Australia; Institute for Health and Sport, Victoria University, Melbourne, Victoria 3011, Australia; Rehabilitation, Ageing and Independent Living Research Centre, Monash University, Melbourne, Victoria 3199, Australia; Faculty of Medicine, Dentistry and Health Sciences, University of Melbourne, Melbourne, Victoria 3010, Australia; National Ageing Research Institute, Royal Melbourne Hospital, Melbourne, Victoria 3050, Australia; National Ageing Research Institute, Royal Melbourne Hospital, Melbourne, Victoria 3050, Australia; School of Public Health, and the Charles Perkins Centre, University of Sydney, Sydney, NSW 2006, Australia; Physiotherapy Department, Melbourne School of Health Sciences, The University of Melbourne, Melbourne, Victoria 3010, Australia; Rehabilitation, Ageing and Independent Living Research Centre, Monash University, Melbourne, Victoria 3199, Australia; National Ageing Research Institute, Royal Melbourne Hospital, Melbourne, Victoria 3050, Australia; Department of Social Inquiry, La Trobe University, Melbourne, Victoria 3086, Australia; National Ageing Research Institute, Royal Melbourne Hospital, Melbourne, Victoria 3050, Australia; Curtin School of Allied Health, Faculty of Health Sciences, Curtin University, Perth, WA 6845, Australia; enAble Institute, Faculty of Health Sciences, Curtin University, Perth, WA 6845, Australia; Brightwater Research Centre, Brightwater Care Group, Perth, WA 6052, Australia; Rehabilitation, Ageing and Independent Living Research Centre, Monash University, Melbourne, Victoria 3199, Australia

**Keywords:** age-friendly, Seniors Exercise Park, physical activity, older people

## Abstract

Physical inactivity among older adults remains a significant public health issue. Age-friendly active spaces with the Seniors Exercise Park equipment in local parks offer an accessible, low-cost opportunity to promote health through physical activity. The ENJOY IMP-ACT project was a multi-site implementation initiative aimed at scaling up the use of the Seniors Exercise Park across six sites in Victoria, Australia. This paper examines factors influencing implementation and uptake. A descriptive practice-based implementation insights approach was used to evaluate the ENJOY IMP-ACT project. Data sources included staff field notes, meeting minutes and park-based activity tracking. Insights were categorized using the TERM framework: training, engagement, resource development, and marketing. Additional analysis considered environmental and organizational factors affecting delivery and uptake. Coordinated planning with local government staff, flexible timelines, and tailored training delivery supported effective program implementation. Local governments with dedicated project officers, clear internal processes, and targeted community engagement strategies demonstrated higher levels of participation. Site visibility, access to amenities, and proactive promotion influenced park usage. Organizational factors, such as changes in staff or limited resources, created barriers to continuity. Efforts to adapt to local contexts, such as modifying training models or leveraging community events, were also important for success. The ENJOY IMP-ACT project highlights the value of a flexible, multi-components implementation framework supported by local partnerships. Consideration of both environmental and organizational factors is essential for sustained delivery of age-friendly health promotion initiatives. These findings offer actionable insights to guide future scale-up efforts in community settings.

*Trial registration* This trial is prospectively registered with the Australian New Zealand Clinical Trials Registry. Trial registration number ACTRN12622001256763. Date registered 20/09/2022. https://www.anzctr.org.au/ACTRN12622001256763.aspx

Contribution to Health Promotion

- This study adds real-world insights into implementing a community-based physical activity program for older adults using outdoor exercise equipment.
- It demonstrates how local governments can adapt and sustain age-friendly initiatives through flexible, practice-informed strategies.
- It highlights environmental and organizational factors that influence implementation success across diverse sites.
- Findings support the use of a structured, multi-component framework to guide scale-up and local adaptation of physical activity programs for older people.

## Background

Physical inactivity among older adults remains a public health challenge, contributing to reduced function, increased falls risk, and poorer overall health (Ding *et al*. 2016, McPhee *et al*. 2016). Age-friendly environments that promote safe, inclusive, and accessible physical activity options for older people have gained growing attention as a potential strategy to support healthy and active aging. Safe use of outdoor exercise equipment designed for older people, such as the Seniors Exercise Park, can result in physical, functional, and social health benefits in community-based settings (Sales *et al*. 2017, 2018, Levinger *et al*. 2020, 2021a, 2021b, 2022b, Brusco *et al*. 2023). This offers the opportunity to engage in physical and incidental activities in local parks to maintain older people’s health at no personal cost.

The usage of outdoor exercise equipment to increase population physical activity levels has been reflected in the growing number of outdoor gyms installed by local governments. However, to maximize the usage of outdoor exercise equipment, scaling up evidence-based physical activity interventions is needed (Jansson *et al*. 2025). Research into physical activity scale-up trials demonstrated that often the effects of physical activity interventions were smaller than reported in pre-scale efficacy trials (Lane *et al*. 2021). Poor adaptation to local community contexts, structural implementation barriers, and lack of implementation strategies and planning have been identified as key factors influencing implementation success (Koorts *et al*. 2018, 2021, Pelletier *et al*. 2020). Systematic reviews of community-based physical activity initiatives indicate that real-world translation is consistently hindered by operational barriers, including competing organizational priorities, limited resource allocation, and a lack of specialized training for delivery staff (Cooper *et al*. 2021). Similarly, the Choose to Move program in Canada demonstrated that flexible implementation approaches, adaptation to local contexts, and strong partnerships with key stakeholders were critical for successful scale-up across diverse community settings (Nettlefold *et al*. 2023). Despite growing evidence regarding the effectiveness of physical activity interventions, there remains limited knowledge about how age-friendly outdoor physical activity initiatives can be successfully implemented, adapted, and sustained across diverse community and local government settings. Consequently, generating evidence regarding how local governments can systematically adopt, manage, and sustain these initiatives within real-world constraints is a vital pre-requisite to successful population scale-up. Understanding the factors that support or hinder implementation is essential to inform future scale-up and long-term sustainability.

**Figure 1:**
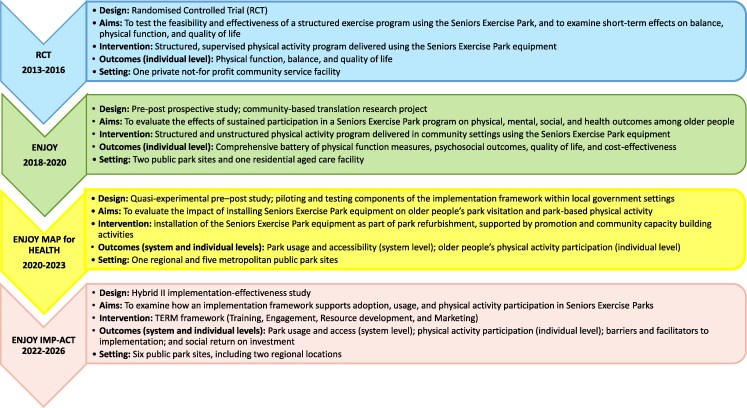
The ENJOY research translation pathway. ENJOY - Exercise interveNtion outdoor proJect in the cOmmunitY; ENJOY MAP for HEALTH - Exercise interveNtion outdoor proJect in the cOmmunitY for older people-More Active People for HEALTHier communities; IMP-ACT - IMProving older people’s health through physical ACTivity.

Community-wide programs have been identified as one of the most effective investments for reducing global physical inactivity (Milton *et al*. 2021). Few outdoor physical activity interventions and promotions have been tested and implemented in community settings and public spaces in Australia. Examples of community-based interventions include the 10 000 steps Australia (Duncan *et al*. 2018) (daily walking steps promotion), ecofit (Jansson *et al*. 2024) (outdoor gym resistance and aerobic training intervention using smartphone technology), and the ENJOY program (Exercise interveNtion outdoor proJect in the cOmmunitY using the Seniors Exercise Park equipment for older people) (Levinger *et al*. 2025b). The ENJOY program of research (Fig. 1), with a primary focus on older people, has progressed from efficacy testing (Sales *et al*. 2017, 2018) to community translation (Levinger *et al*. 2020, 2021b), implementation framework development (Levinger *et al*. 2024b), and implementation scale-up within local government contexts (Levinger *et al*. 2024c, 2025b) (ENJOY IMP-ACT: IMProving older people’s health through physical ACTivity). The ENJOY IMP-ACT project is unique due to its collaborative approach, which actively involves local councils and community members in its delivery, and its targeted focus on older people, a population often under-represented in outdoor physical activity infrastructure initiatives.

The ENJOY IMP-ACT project was a multi-site implementation initiative designed to support the scale-up of the Seniors Exercise Park use through the TERM framework (Training, Engagement, Resource development, and Marketing). This framework was developed through our previous research and is informed by the Interactive Systems Framework and ecological models, which highlight the importance of translating evidence into practice through community-centered approaches and active stakeholder engagement (Durlak and DuPre 2008, Wandersman *et al*. 2008, Levinger *et al*. 2021a). The TERM framework evolved iteratively from earlier ENJOY projects where several implementation components were piloted in partnership with local governments. During that phase, discrete elements such as training delivery, volunteer engagement, resource development, were tested to support park activation. ENJOY IMP-ACT represents the first comprehensive application of the full TERM framework across multiple sites, enabling systematic evaluation of its utility in supporting adoption, scale-up, and sustainability. The intervention increased park usage and physical activity participation among older adults across six Victorian communities, with a four-fold increase in the number of older adults using the Seniors Exercise Park and a two-fold increase in the number engaging in physical activity compared with baseline (Levinger *et al*. 2025b). However, real-world implementation revealed variation in local adoption, program delivery, and community engagement across sites. These site-specific differences provide valuable insights into the practicalities of embedding and sustaining age-friendly physical activity infrastructure and support programs over time within diverse local government, community, and environmental contexts.

This paper examines factors that influenced the implementation and uptake of the ENJOY IMP-ACT intervention. It presents practice-based implementation insights (Wensing and Wilson 2023) from the ENJOY IMP-ACT project, which aimed to deliver a community-based older adult physical activity initiative at scale across six local government areas using the TERM framework (Levinger *et al*. 2024c). The purpose of this paper is to examine variations in program delivery and their potential implications for implementation outcomes, based on two outcomes that reflect the success of the project: (i) the level of community usage, specifically older adult participation in physical activity using the Seniors Exercise Park, and (ii) the continuation of sessions and park activation activities by local governments and trained community volunteers following the conclusion of the formal intervention.

Drawing on site-level process data, local government feedback, and environmental context information, we identify facilitators, barriers, and critical learnings that may influence program delivery and sustainability. Using a practice-based implementation insights approach (Wensing and Wilson 2023), we identify real-world examples that contribute to an understanding of how the TERM framework was locally adapted and the external factors (environmental and organizational factors) that may have influenced project success. The findings aim to guide future scale-up efforts and provide practical strategies for delivering age-friendly physical activity initiatives through partnerships with local government and communities.

This paper presents practice-based implementation insights derived from a descriptive and interpretive analysis of project documentation, implementation tracking data, and partner feedback.

## Methods

### Study context and intervention

Five local governments/municipalities (six public sites/parks that had the Seniors Exercise Park outdoor exercise equipment) underwent a 3-month control period followed by 9-months implementation intervention (TERM framework intervention: Training, Engagement, Resources development, Marketing and promotion), and a maintenance phase (3 months) (Levinger *et al*. 2024c). The 3-month maintenance phase was included to assess the short-term continuation of park activation activities and community engagement following the withdrawal of formal implementation support. The “TERM” framework components were focused on building capacity, knowledge, upskilling, and engagement aimed at increasing usage and uptake of physical activity using the Seniors Exercise Park (Levinger *et al*. 2025b). The Seniors Exercise Parks incorporate a range of exercise stations designed to improve older peoples’ balance, strength, function, coordination, joint movement, mobility, and dexterity. Full details of the methodology are reported in the protocol paper (Levinger *et al*. 2024c). The study was approved by the Monash University Human Research Ethics Committee, Melbourne Australia (Project ID: 35502). Verbal or written consent was obtained prior to participation.

### Data sources and collection

This practice-based implementation insights paper draws on practical observations and site-level activity data and audits to identify examples of implementation approaches that may have supported or hindered project delivery and successful outcomes. Environmental factors (such as site location, park features, amenities, and accessibility) and partner organizational factors (internal processes and operation) were also reviewed and discussed. Data were collected by the research staff through systematic auditing of project-related activities and implementation processes. The number of park-based sessions (e.g. volunteer-led sessions), events and activities delivered, along with participant attendance, were documented, and local government-reported records of scheduled programming during the 9-month implementation and 3-month maintenance periods were collated.

#### On-site park observation

Structured park observation audits using the System for Observing Play and Recreation in Communities (SOPARC) (McKenzie *et al*. 2006) were also collected to document individuals who walked by (did not interact or use the equipment) within the vicinity of the Seniors Exercise Park (the equipment and the immediate surrounding space, including adjacent facilities/amenities/areas where visitors might interact). Observation periods were conducted over 10 day periods at 7 time points across the project (70 observation days per park site), with 14 scans per day comprising 10 minute observations every 30 minutes during the early morning (07:30–10:30), mid-day (12:00–13:30), and late afternoon (15:00–17:30). Further details of the observation methods are provided elsewhere (Levinger *et al*. 2024c, 2025b). Several research staff trained in the SOPARC methodology conducted the park audits. A project manual/protocol was also used to ensure consistent data collection. These park audits provided an indication of site-level foot traffic and the visibility of the park to passers-by and other park users.

#### Digital engagement and promotion monitoring

Data on usage of the MY ENJOY Health^®^ mobile application were also collected from the commencement of the intervention period to the completion of the maintenance period using an online platform (Metabase). The MY ENJOY Health^®^ mobile application was developed to support use of the Seniors Exercise Park and provided users with exercise instructions, workout programs, exercise videos, and safety information. Quick response (QR) codes were installed on the various exercise stations to provide direct access to exercise instructions and videos, as well as links to download the mobile application. Data on app registrations and QR code scans were collected as indicators of digital engagement with the intervention. In addition, the mode and frequency of promotion and marketing (e.g. social media channels) were extracted through monthly online audits conducted by a member of the research team. These audits involved reviewing local government communication channels, including council websites, Facebook pages, and other publicly accessible social media platforms, to identify and document promotional activities related to the Seniors Exercise Park and ENJOY IMP-ACT project. An Excel template was created by the research staff to assist local government staff and ensure consistent recording of promotional activities across sites. Information identified through the online audits was entered into the template by the research staff, and local government staff were contacted monthly to verify the information and provide details of any additional promotional activities that were not publicly available or could not be identified through the online searches. Additional implementation insights were sourced from research and project meeting minutes, research staff field notes, and internal tracking of scheduled community induction sessions, volunteers training sessions, promotional events, and informal activations. These data were collected prospectively throughout the project by members of the research team as part of routine project monitoring and implementation support activities. Field notes documented observations relating to program delivery, stakeholder engagement, barriers, facilitators, adaptations, and operational issues encountered during implementation. Meeting minutes and implementation records were reviewed retrospectively to identify additional examples and contextual factors that may have influenced project delivery and uptake.

### Synthesis of implementation insights

Data collected and reported here were categorized according to the four components of the TERM framework. The categorization process was undertaken collaboratively by members of the research team, who reviewed information from all data sources and mapped relevant findings to the TERM framework components. Environmental and organizational factors identified through project documentation, field notes, meeting records, and site audits were also reviewed. Through this approach, we identified illustrative examples, both facilitators and challenges, within each TERM component and the environmental and organizational factors that appeared to influence usage of the park and sustainability. These practice-based insights were synthesized narratively to inform key learnings and actionable recommendations for future implementation efforts.

## Results

A synthesis of main activities in each TERM component and the environmental and organizational factors is provided below. A summary is also provided in Table 1, which includes key examples, learnings, and recommendations.

**Table 1:** A summary of key learning and recommendations as well as illustrative examples under each of the TERM component.

Framework component	Method of data collection	Key learning and recommendations	Illustrative examples
Training—knowledge transfer and capacity building activities Train the trainer: Seniors Ambassadors/Champion Allied Health Professionals Induction sessions	Research staff audit files Online registration and attendance record Attendance sheet records collected by research staff on-site	Order of activities and events Early and well-sequenced training—beginning with community volunteers—helps build program momentum, ensures support for community engagement, and reduces the risk of drop-off in participation or volunteer capacity due to delays or extended breaks Volunteers’ recruitment, process and management Clear role expectations during volunteer recruitment and maintaining a manageable cohort size (6–8) aligned with programming needs are key to sustaining volunteer engagement and preventing burnout. Offering a second round of volunteer training too early may not suit local needs; adopting a flexible, interest-driven approach, such as mentoring new recruits through experienced volunteers, can support sustainability without overwhelming local government capacity. Strategic recruitment and training of volunteers from seniors groups/organizations can lead to member-based programs	“Come and try” Session with a local Retirement Village was organized at the completion of the training while no local government-led sessions were yet available following the event for interested participants. Sessions were commenced 5 weeks later which led to low number of attendees. A volunteer from local government dropped out at the completion of the training due to concerns over data/privacy as part of onboarding volunteer process (e.g. a police check), which was introduced only after training completed. Low number of volunteers recruited at two of the sites (four per site), with three that have discontinued in one site. Only one volunteer remained in one of the sites. Only three additional volunteers in total were trained at two sites in the second round of training. Training two volunteers from seniors’ group/organizations (such as University of the Third Age (U3A)) led to formation of exercise programs run by the organization to its members. While allied health professionals participated in the workshop training, their ongoing involvement at the parks and referrals to induction sessions remained limited.
Engagement Community events “Come and try” sessions Local/project Advisory committees	Local government-reported records Meeting notes Email correspondence with local governments	One to two large community events per year aligned with annual themed celebrations (e.g. Seniors Week, Falls Prevention Month) can boost participation and effectively leverage existing local government resources dedicated to these events. Ongoing engagement with local groups, supported by local advisory committees where feasible, can strengthen community participation and promote long-term program success	Open Day event run by a local government (also promoted by staff from the local community health center) led to a large number of attendees and an increase in the number of participants in the weekly come and try sessions following the event. The event was promoted on a local newsletter distributed to all residents. Closure of one of the parks for a few months led to a drop in attendees and loss of momentum of the weekly come and try sessions. One local government formed a local advisory committee—comprising local health professionals and community representatives which led to the establishment of an independent structured exercise program led by a qualified instructor at the site.
Resource development On-site QR code and mobile app On-site Instructional signage Instructional flyers	Online data platform (Metabase) for MY ENJOY Health^®^ mobile application Research staff field notes	Combining digital tools with simple on-site and print-based resources ensures broader community reach by accommodating varying levels of technological skill and personal preference.	Creation of simple flyers assisted usage of the mobile app and the outdoor equipment Local government-specific dedicated webpage and resources assisted with community awareness and promotion
Marketing and Promotion Various platforms: online/offline	Regular online searches Local government staff reports Staff field notes for promotional events and resources	Tailoring promotion through age-specific networks and using traditional methods such as printed flyers alongside digital channels enhances reach and engagement among older adults and should be prioritized over just using generic social media strategies	Lack of promotion of a community event in one local government (due to internal restrictions) led to a very low number of attendees. When only generic social media platform used (such as local governments’ Facebook), there was limited effect on older people attendance at events or sessions. A local government that demonstrated the highest promotion frequency and widest reach experienced strong community demand and attendance, resulting in the establishment of three weekly peer-led sessions.
Overarching recommendation		Early collaborative planning within local government and across local government departments is essential to align timelines, logistics, and local procedures, ensuring smoother implementation and responsiveness to site-specific needs	

### Intervention TERM framework

Training (knowledge transfer) included training of allied health professionals (a half day workshop per local government) and community volunteers (5-weeks train the trainer module) to support engagement and ongoing usage of the Seniors Exercise Parks, based on our previous work (Levinger *et al*. 2022a, 2024b). In addition, up to 12 community induction sessions with qualified research staff (e.g. accredited exercise physiologist or physiotherapist) were delivered at each site, to provide extra support for park users who require additional supervision. Thirty-eight allied health professionals (including other health-related occupations) attended the workshop training and 37 community volunteers (champions/ambassadors) completed the train the trainer modules across the six sites (4–8 volunteers per site). A total of 69 community induction sessions were delivered across the six sites.

The timing and sequencing of training delivery played a critical role in engaging the community and sustaining momentum around park-based activities. Having trained volunteers available prior to commencement of allied health training and induction sessions was important, as they provided the necessary support to run events and activities that engaged older adults. Training volunteers enabled the establishment of free, weekly, peer-led sessions (“come and try”) at each site during both the intervention and maintenance phases. Extended breaks or gaps between training and program delivery, such as during holidays, resulted in loss of interest, reduced engagement, and diminished retention of practical skills among volunteers.

An additional round of community volunteer training was offered to all local governments toward the end of the intervention phase of each site. While most sites did not require further training due to an adequate number of active volunteers or limited community interest, two sites proceeded with additional training. In response to further interest at those sites, a modified training model was developed and delivered to three new community members. This approach combined mentorship from experienced volunteers with practical support and supervision from the research team. Delivering a second full training program across all sites was deemed unnecessary within the study timeline and may be more appropriate at a later stage, depending on volunteer turnover and local program needs.

Volunteer recruitment and management varied across local governments due to differing local practices. Local governments that set clear expectations regarding the role and responsibilities of volunteers early in the recruitment process, ideally before the commencement of training, had reduced dropout (Levinger *et al*. 2025a). The recruitment of a sufficient number of volunteers (∼6–8 per site) allowed for attrition or turnover, while also remaining manageable for local government staff. The alignment of this number with the weekly programming schedule, while also building in breaks to prevent fatigue and burnout, helped to ensure all volunteers had opportunity to participate meaningfully.

Connection and engagement with allied health professionals during the training workshops was useful in establishing connections with local health providers; however, it resulted in limited direct involvement of allied health professionals in delivery of programs or referral of clients. A lack of time, limited resources to support clinicians, and the geographical distance of the parks from clinics or community health centers appeared to contribute to this reduced engagement. There were a total of 10 referrals from health professionals to induction sessions across sites. Five of these were at one site and referred by a health professional using the equipment with an exercise group established in collaboration with the local government; this provided time and resources for connection with the community park sessions. Further research is needed to identify effective strategies for supporting allied health professionals to integrate park-based activities into their practice and increase utilization of the Seniors Exercise Parks.

Engagement was integrated at both the project governance level, through formal agreements and a Community of Practice committee, and at the community level via local advisory committee, come and try volunteer-led sessions, and local events such as open days, Seniors’ Week events, and Community Health Expos.

All local governments signed an agreement prior to project commencement and assigned a key project officer who participated regularly in project meetings and activities. Engagement with local community groups, seniors clubs, and community health centers often facilitated broader reach and increased community participation, particularly when such groups actively promoted the initiative to their members. However, in many instances, these initial connections were not followed up or maintained, limiting longer-term engagement potential.

Although local governments were encouraged by the research team to establish local advisory committees to support community input and guide program delivery, only one local government implemented this recommendation. This committee, comprising local health professionals and community representatives, met every 4–6 weeks and provided a mechanism to support the delivery of an independent structured exercise program (led by a qualified instructor) which had been established at the site. Other local governments either lacked the capacity or did not perceive an immediate need to form such a committee.

Come and try community sessions held per site ranged from 13 to 57 in the intervention period and 8–26 in the maintenance period, with total number of participants per site ranging from 34 to 220 and 12 to 124 in the intervention and maintenance periods respectively. A total of eight community events were held throughout the project. Large-scale community events that were well-promoted across multiple platforms led to strong attendance and helped reinvigorate park-based activities over time. For example, at one site, following a large community event, community session attendance increased from an average of 17.5 participants per month (in the 2 month period prior to the event) to an average of 28 participants per month (following the event to the end of the project period).

Resource development included a mix of on-site information (such as instructional signage), printed flyers, on-site QR codes, and the My ENJOY Health^®^ mobile application (https://www.nari.net.au/enjoy-me-app) to assist with familiarization with the outdoor equipment and its safe usage. There were 165 registered users for the My ENJOY Health^®^ mobile application across project sites in the implementation and maintenance periods. Three sites had significantly higher numbers of registered users; these sites had increased community engagement through promotional events or more frequent come and try sessions (delivered 2 to 3 times/week for part of the project period). The QR codes located at each exercise station at the Seniors Exercise Parks provided the users with an instant easy access to view exercise resources; there were 3702 QR codes scanned at Seniors Exercise Parks across the project period (although these scans included other park locations). The workshops and trainings were also complemented with supportive printed booklets and manuals. All local governments created dedicated webpages, with some collecting professional footage (e.g. photos) and developing site-specific resources (e.g. post-cards) to support usage and promotion of activities.

### Marketing and promotion

Marketing and promotion efforts were implemented by all local governments using social media and other platforms, though frequency and methods varied. Across the intervention period, local governments promoted the Seniors Exercise Park program between 8 and 82 times per site over a span of 2–9 months. Promotion efforts primarily aimed to advertise “come and try” days and typically involved four to nine different communication channels. The most used platforms included Facebook, print news, and e-newsletters (each used by all five local governments), followed by Instagram, webpages, emails, and printed flyers. During the maintenance period, promotion ranged from 0 to 15 instances per site over 1–3 months, primarily focused on “come and try” activities, using between 1 and 6 promotional channels. Sites that demonstrated the most consistent and frequent promotional efforts—marked by a high number of promotional activities, extended promotional timelines, and diverse communication channels—were also those where the program gained the most traction. For example, one such site, with the highest promotion frequency and broadest reach, established three weekly peer-led sessions due to high demand and consistently large community attendance.

Reliance on generic social media channels such as Facebook often limited reach, particularly among older adults. Greater impact was observed when promotional efforts were tailored to the target demographic—for example, through aging-specific newsletters, distribution lists, and seniors’ networks. Traditional methods such as printed flyers, physical newsletters, and displays in libraries or community centers also proved essential for reaching older people who may not engage with online platforms.

### External factors

**Figure 2:**
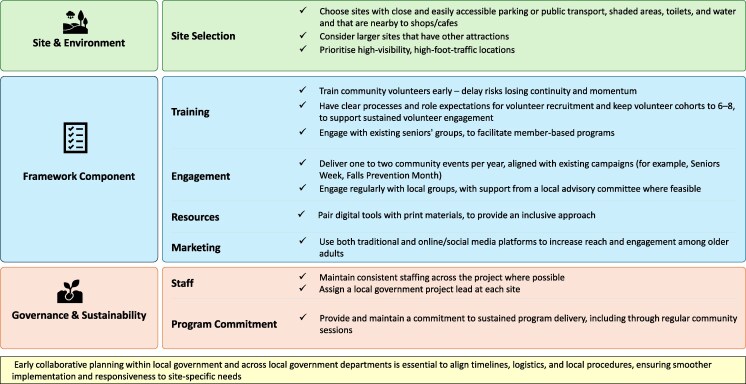
ENJOY IMP-ACT project: summary of key implementation recommendations for site selection, program delivery, and governance and sustainability.

Environmental factors, such as site location, park features, amenities, and accessibility, and partner organizational processes, while outside the direct control of the research team, were important to consider, as they may have directly influenced park usage and the sustainability of park-based activities beyond the formal project period. A summary of key aspects of the environmental and organizational factors are provided in Tables 2 and 3 respectively. Figure 2 provides a summary of the key implementation recommendations for site selection, program delivery, and governance and sustainability.

**Table 2:** Park access, amenities, features, and location.

	Barry Road Community Activity Center, Thomastown	Park Andrew Pocket Park, Eltham	Donald Mclean Reserve, Spotswood	Central Park Community Center, Hoppers Crossing	The Elms Reserve, Kilmore	Chittick Park, Seymour
Park type^a^	Pocket park	Pocket park	Large park	Pocket Park	Pocket park	Large park
Access	Allocated parking Public transport within 400 m	Allocated parking Public transport within 400 m	Allocated parking Public transport within 400 m	Allocated parking Public transport within 1 km	Street parking Public transport within 400 m	Allocated parking Public transport within > 1 km
Amenities	Shade sail Toilets (within the community center) Adjacent community center Adjacent roofed/shaded picnic area No water fountain	Shade sail Toilets Adjacent roofed/shaded picnic area Water fountain	No cover Toilets Adjacent roofed/shaded picnic area Water fountain	Shade sail Toilets (within the community center) Adjacent community center No water fountain	No cover No toilet Adjacent roofed/shaded picnic area No water fountain	No cover Toilets (across the road) Adjacent roofed/shaded picnic area Water fountain
Location	In a residential area Nearest cafes/shops >1 km Adjacent to community center and sports court Picnic/BBQ area Small playground area	Close to suburban center Nearby to cafes/shops Adjacent to sports courts Picnic/BBQ area Small playground area Walking track	Close to suburban center Nearby to cafes/shops Adjacent to sports fields Picnic/BBQ area Large/multiple playground areas Walking track	In a residential area Nearest cafes/shops ∼1 km Adjacent to community center and small sports field Picnic/BBQ area Multiple playground areas Internal walking track	In a residential area Nearest cafes/shops >1km Adjacent to retirement village Picnic/BBQ area Small playground area Walking track	Close to suburban center Nearby to cafes/shops Adjacent to sports fields Picnic/BBQ area Multiple playground areas
^b^Visibility and foot traffic	Medium visibility Low foot traffic	High visibility High foot traffic	High visibility High foot traffic	Medium visibility Medium foot traffic	Low visibility Low foot traffic	High visibility High foot traffic
^c^Pass-by foot traffic	317 people	1034 people	3403 people	405 people	330 people	802 people

^a^Park types—Pocket park is defined as a small park, localized space with limited recreational activities. Large park is defined as large park that offers a wider range of recreational activities.

^b^Visibility refers to how easily the Seniors Exercise Park can be seen from the surrounding streets, pathways, or nearby public facilities.

^c^Pass-by foot traffic refers to number of people observed to move past the Seniors Exercise Park equipment area, without interaction.

**Table 3:** Local government organizational structures and engagement.

Local government organizational structures/engagement	Site 1	Site 2	Site 3	Site 4	Site 5 and 6
Number of local government staff contributing to project	2	2	2	1	2
Local government department/unit responsible for the Seniors Exercise Park program	Ageing well	Community Support Services	Sports and Recreation	Sport and Recreation	Community Development and Youth Services
Direct local government officer to manage project and support volunteers	Positive Ageing Officer	Positive Ageing Officer	Sports and Recreation Officer	Inclusion and Participation Officer	Community Development Officer—Life stages and Inclusion
Staff changes across project	—	—	Three staff change	—	—
Regular participation in project and team meetings	√	√	√	√	√
Local government commitment to maintain the volunteers-led program	√ Weekly sessions during school terms in Spring and Summer	√ Once to twice weekly sessions (pending volunteers availability and capacity to assist)	√ Weekly sessions	√ Two to three weekly sessions	√ Aim to transition in 2025 to a community-led model in partnership with a community group that would support governance/administration of the volunteer program.

#### Environmental factors—the built environment, park features, and surrounding areas

The design, location, and access of the Seniors Exercise Park within recreational parks can facilitate usage and access by older people (Levinger *et al*. 2025c). The locations and features of the six parks varied; a summary of these characteristics is provided in Table 2. Parks with high visibility (i.e. how easily the Seniors Exercise Park can be seen from surrounding streets or public areas), nearby amenities (such as toilets, shade, and cafés), larger size and attractions, and good access to public transport and parking (as seen in Eltham, Spotswood, and Seymour suburbs/locations) recorded higher levels of foot traffic, as measured through SOPARC observations of people passing through the vicinity of the Seniors Exercise Park, and generally demonstrated greater community engagement (see Table 2). These site-level observations suggest that visibility, location, and supportive infrastructure may have influenced awareness of and engagement with the Seniors Exercise Parks. In contrast, parks with lower visibility, such as Kilmore, may see reduced engagement, particularly among older adults who prefer easily accessible and socially visible locations.

#### Partner organizational factors (internal processes and operation)

We have previously identified that the operational structures and internal processes of local governments play a key role in enabling research partnerships, supporting engagement with the research team, and facilitating smooth delivery of project activities (Levinger *et al*. 2021a). Similarly, the continuation of volunteer-led community sessions, essential for sustaining older adults’ engagement in park-based physical activity, depends heavily on ongoing support from local governments (Levinger *et al*. 2025a).

Across participating local governments, we observed notable variations in operational practices, including approaches to volunteer recruitment, promotional strategies, and the specific departments or units responsible for overseeing the Seniors Exercise Park program (see Table 3). Changes in key local government staff during the project often disrupted program continuity, requiring re-engagement with the research team, reorientation to the project, and rebuilding relationships with existing volunteers. These transitions introduced delays and impacted the momentum of ongoing activities. Conversely, a common factor contributing to program success across sites was the presence of a dedicated local government officer, supported by allocated local government resources and a commitment to sustaining the program beyond the study period. Despite the difference in the local government department/unit that was responsible for managing the project, all direct staff attended and actively participated in internal and project meetings. Local governments that demonstrated long-term intent and provided consistent staffing were better positioned to integrate and maintain the Seniors Exercise Park program as part of their broader age-friendly community initiatives.

## Discussion

Scaling up health promotion interventions, such as community-based physical activity, is inherently complex where successful scale-up depends on dynamic interactions between organizational capacity, stakeholder perceptions, contextual factors, and adaptive processes (Koorts *et al*. 2021). Integrating practice-based knowledge into implementation frameworks is valuable for bridging the gap between research and real-world application. The pragmatic design of the ENJOY IMP-ACT project, incorporating the TERM framework, demonstrated how multiple, interconnected components can support collaborative efforts to enhance community participation. Within this complex implementation context, the combined contribution of these components translated into measurable outcomes, with a four-fold increase in the number of older park users reported following the intervention. In addition, a significant increase in the number of older people engaging in physical activity using the Seniors Exercise Park was observed post-implementation (Levinger *et al*. 2025b), further demonstrating the positive impact of this multi-component, partnership-based approach.

Comparable community-based research initiatives, such as the Healthy Ageing Promotion Programme for You (HAPPY) in Singapore (Merchant *et al*. 2021), have demonstrated similar implementation dynamics to those observed in ENJOY IMP-ACT. The HAPPY trial delivered structured dual-task exercise and social engagement activities across multiple community sites using partnerships with local organizations and trained volunteer leaders (Merchant *et al*. 2021). Despite differences in intervention content, HAPPY’s implementation approach was similar to that of ENJOY IMP-ACT, including engaging community champions, leveraging existing social networks, and embedding activity within familiar community settings. These similarities reinforce the value of implementation frameworks that accommodate complexity and iterative adaptation when promoting physical activity among older populations in real-world contexts.

While the research team led the delivery of the various training components within the TERM framework, advance planning meetings with local government staff were essential to establishing realistic timelines, determining the optimal sequence of activities, and clarifying volunteer recruitment requirements. Early and well-sequenced community volunteer training, delivered before allied health training, induction, and community sessions, is likely to assist in establishing ongoing peer-led activities and sustain community engagement. A flexible approach that includes clear role expectations, manageable group sizes, and the option for later mentorship-based training can support volunteer retention and long-term program delivery. Allied health engagement in park-based initiatives may be strengthened through ongoing relationship-building and practical support between local governments and local health care agencies or physical activity providers. This was demonstrated at one site, where an exercise group at the Seniors Exercise Park delivered by a health professional with local government support led to increased referrals to community sessions. Strategies such as increasing awareness of the Seniors Exercise Park among local health professionals through targeted communication and promotional activities, providing clear referral pathways, and fostering partnerships between local governments and healthcare providers may further support referral and participation. Addressing potential barriers such as time, resources, and proximity may be required to ensure their sustained involvement and client referral.

Strong and sustained engagement with local groups and community organizations can enhance program visibility and drive participation. While most local governments chose not to form local advisory committees, the site in which such a committee was formed found it useful in facilitating local program delivery. According to the local government staff and project observations, the committee supported communication, coordination, and planning of program activities, although its direct contribution to implementation outcomes could not be determined independently of the broader implementation activities. While further evaluation is needed to understand their specific contribution, local governments may consider forming local advisory groups, where relevant and feasible, to support relationship-building, communication, and local program delivery. In addition, using both digital tools and traditional resources allowed broader community reach, accommodating varying levels of digital literacy and enabling individuals to engage through their preferred format. Targeted, multi-channel marketing strategies are recommended to ensure broader and more inclusive community reach with frequent regular promotion. Hosting one to two community events per year is recommended, ideally aligning with established health awareness periods or aging-related yearly theme celebrations such as Seniors Week or Falls Prevention Month, to maximize visibility and relevance. While community events appeared to increase visibility and participation, they also required considerable planning, staff time, volunteer support, and organizational coordination. Local governments should therefore consider resource requirements and capacity when planning large-scale activation events and ensure appropriate follow-up opportunities are available to support ongoing participation after initial engagement. Overall, the collaborative engagement process employed in the TERM framework enabled the adaptation of the project to local government-specific internal procedures, logistical constraints, staff capacity, and weather-related factors. Early alignment between the research team and local government partners ensured smoother implementation and greater responsiveness to local operational contexts.

Although external factors such as the built environment and organizational structures were outside the control of the project team, they remained critical for future consideration. Weather and seasonal variation may also influence participation in outdoor physical activity and use of outdoor exercise spaces (Garriga *et al*. 2021, Levinger *et al*. 2024a). While these factors were outside the control of the project team, seasonal effects were accounted for in the effectiveness analysis of the ENJOY IMP-ACT project (Levinger *et al*. 2025b). Parks with high visibility, nearby amenities and features, and good access may attract more spontaneous use/walk-up participation and foster a sense of safety and community presence, similar to other studies (Giles-Corti *et al*. 2005, Sami *et al*. 2020, Bartels *et al*. 2023). In contrast, parks with lower visibility may benefit from regular, scheduled programming or organized group activities to drive awareness, encourage participation, and support ongoing engagement among older adults. Importantly, infrastructure alone may not be sufficient to increase access or usage of outdoor exercise equipment (Leavy *et al*. 2022, Paudel *et al*. 2024). The importance of visibility, colocation with amenities, active promotion, and social support was further reflected in qualitative findings from community stakeholders involved in the ENJOY IMP-ACT project, who identified these factors as key facilitators of park utilization, while low awareness and poor visibility were perceived as barriers to engagement (Muoio *et al*. 2026). Consistent staffing, clear internal processes, and dedicated local government support are essential for sustaining community-based programs; local governments should allocate a dedicated project officer and plan for staff continuity to ensure program momentum and volunteer engagement are maintained (Levinger *et al*. 2025a). Where possible, efforts need to be made to manage or adapt to these external factors to mitigate their impact on program delivery. For example, differences in site visibility, amenity access, or internal staffing changes may be addressed through tailored strategies to maintain program engagement. These may include greater promotion to group sessions and community activations at less visible sites, leveraging local events and community networks to raise awareness, partnering with walking groups or seniors’ organizations to incorporate the park into regular activities, and documenting key program procedures and contacts to support continuity during staff transitions. The findings and observations can be interpreted through several established behavioral and implementation frameworks. First, consistent with the Socio-ecological model, uptake and sustainability of the Seniors Exercise Park programs and activities were likely influenced by interacting factors across individual, interpersonal, organizational, and community levels (Sallis *et al*. 2015). When supportive conditions aligned across the different levels, sites with organizational capacity and proactive community engagement created reinforcing conditions that enabled participation. Second, these observations could also be further supported through the Consolidated Framework for Implementation Research (Damschroder *et al*. 2022), particularly the importance of inner setting readiness, engagement processes, and local adaptability of the intervention. Our findings are consistent with previous research examining implementation of community-based interventions, which identified organizational structures, staffing capacity, stakeholder engagement, resource availability, implementation planning, and adaptation to local contexts as key determinants of successful implementation and scale-up (Cooper *et al*. 2021, Murphy *et al*. 2023). Similarly, the ENJOY IMP-ACT highlighted the importance of dedicated local leadership, community partnerships, volunteer engagement, and ongoing organizational support in facilitating implementation and sustainability. Finally, from a behavior change perspective, the findings may also be consistent with the COM B model (Michie *et al*. 2011), whereby community participation occurred when capability was enhanced through volunteer-led sessions, opportunity was created through accessible and visible infrastructure, and motivation was reinforced through social support and engagement activities.

This study has several strengths and limitations. Strengths include the inclusion of multiple local government sites and the use of diverse data sources, including implementation records, park observations, activity tracking, and stakeholder feedback. This provided a comprehensive understanding of real-world implementation processes and enabled the identification of contextual factors that may not be captured through traditional outcome evaluations. However, the findings should be interpreted in light of several limitations. The analysis was descriptive and interpretive in nature. In addition, the TERM framework was implemented as an integrated multi-component strategy, and therefore the contribution of individual implementation components to observed outcomes could not be evaluated independently. As a practice-based synthesis, the implementation insights were generated by integrating multiple project data sources rather than through a prospectively designed qualitative evaluation. Overall, the practical lessons learned from real-world experiences in the ENJOY IMP-ACT project offer valuable insights that can inform future implementation of community-based physical activity and health promotion initiatives, particularly those aiming for sustained community-based delivery and local government-led involvement.

## Data Availability

The datasets generated and/or analyzed during the current study are not publicly available due to ethical restrictions but are available from the corresponding author on reasonable request.
